# Five-year results evaluating the healing of intrabony defects following treatment with A-PRF+ or EMD: a randomized controlled trial

**DOI:** 10.1007/s00784-026-06806-x

**Published:** 2026-03-23

**Authors:** Boróka Klára Csifó-Nagy, Bálint Czufor, Eleonóra Sólyom, Ferenc Dőri

**Affiliations:** Department of Periodontology, Faculty of Dentistry, Semmelweis University, Szentkirályi u. 47, Budapest, 1088 Hungary

**Keywords:** Platelet-rich fibrin, Enamel matrix derivative, Intrabony defects, periodontal healing, Long-term studies

## Abstract

**Objectives:**

The aim of the study was to clinically evaluate the long-term healing of intrabony periodontal defects treated with a new-generation platelet-rich fibrin (A-PRF+) compared with enamel matrix derivative (EMD).

**Materials and methods:**

Thirty intrabony defects in 18 patients were randomly assigned to treatment with A-PRF+ (test, *n* = 15) or EMD (control, *n* = 15). Clinical parameters were assessed at baseline, 6 months, 1 year, and 5 years post-surgery. Clinical attachment level (CAL) was the primary outcome variable. Following full-thickness flap elevation, defect debridement, scaling, and root planning were performed. Defects were filled with A-PRF + or EMD according to group allocation and stabilized with sutures. At the 5-year follow-up, 26 defects in 14 patients were available for evaluation.

**Results:**

Both treatment methods resulted in statistically significant PD reductions, respectively CAL gains after 6 months, and the results were maintained 5 years post-operatively. At 5 years no statistically significant differences were found between the two groups as the mean CAL gain was 2.92 ± 1.65 mm in the test group, and 3.84 ± 1.81 mm in the control group, respectively (*p* < 0.05).

**Conclusion:**

Within the limitations of this study, A-PRF+ demonstrated clinical outcomes comparable to EMD in the surgical treatment of intrabony periodontal defects, with stable long-term results.

**Clinical Relevance:**

A-PRF + may represent a reliable autologous alternative for periodontal regeneration, offering favorable and stable clinical outcomes over a five-year period.

## Introduction

Periodontal disease, a chronic multifactorial inflammatory condition, leads to the destruction of tooth-supporting tissues. Comprehensive periodontal therapy aims to stop attachment loss and achieve complete regeneration of the lost tissues. Periodontal osseous defects, especially intrabony defects, are associated with a compromised prognosis of the affected teeth. The regenerative potential of the periodontal tissues are influenced by the morphological characteristics of the defects [1]. Nibali and Cortellini proposed a treatment-oriented classification of periodontal infraosseous defects to facilitate clinical decision-making. Infraosseous defects are categorized as intrabony or interradicular, reflecting their distinct biological and therapeutic characteristics. For intrabony defects, the traditional wall-based classification is supplemented with additional morphological parameters that may influence regenerative outcomes [2].

In recent decades, biological agents have gained, significant attention for promoting periodontal regeneration. Autologous platelet concentrates, widely used in various medical fields for over 20 years, show promising results in fields of periodontology. The primary goal was to develop a treatment approach that effectively utilizes the body’s natural healing capabilities through the use of platelet concentrates, primarily achieved with the aid of growth factors. Growth factors play a significant role in tissue regeneration [3]. The characteristics of platelet-rich plasma (PRP), plasma rich in growth factors (PRGF), and platelet-rich gel (PRG) may be affected by anticoagulants used in their preparation [4]. To overcome this, platelet-rich fibrin (PRF), a second-generation platelet concentrate, was developed by eliminating anticoagulants and employing a modified centrifugation protocol, enabling its use across various medical fields. Over two decades have elapsed since PRF was first introduced [5]. The introduction of leukocyte-rich platelet-rich fibrin (L-PRF) markedly enhanced wound healing and tissue regeneration [5]. Recent advancements in centrifugation protocols, involving reduced time and speed, have given rise to the “low-speed centrifugation concept,” creating new opportunities for improved outcomes [6]. The advanced platelet-rich fibrin (A-PRF), a next-generation PRF preparation, exhibits higher platelet and neutrophil granulocyte concentrations and sustains prolonged growth factor release, similar to standard PRF [7]. The reduced centrifugal force (208 g) enables a more even distribution of leukocytes, unlike earlier protocols with higher G-forces that concentrated leukocytes primarily in the proximal tube region. Furthermore, A-PRF features a less dense fibrin network with larger interfibrous spaces [7]. Histological comparisons reveal that this less dense structure significantly enhances cellular migration into the fibrin matrix, resulting in markedly increased vascularization after subcutaneous implantation in mice [8]. A minor adjustment to the centrifugation protocol produced a novel platelet concentrate, Advanced PRF Plus (A-PRF+). This modified protocol allows the clot to separate easily from the adjacent red blood cell fraction, facilitating immediate application at the surgical site. The fibrin network of A-PRF+ displays porosity comparable to A-PRF, with cells distributed uniformly throughout the clot [9].

New-generation platelet-rich fibrin may further advance periodontal wound healing. Currently, regenerative periodontal therapy achieves partial tissue restoration to a limited extent, while complete periodontal restoration remains idealistic [10]. In this context, Dőri et al. demonstrated in a long-term randomized clinical trial that treatment of intrabony defects with enamel matrix derivative (EMD) and natural bone mineral (NBM), with or without adjunctive platelet-rich plasma (PRP), yielded significant clinical improvements maintained over 5 years; however, PRP provided no additional benefit compared with EMD + NBM alone [11].

Enamel matrix derivative (EMD), introduced over 20 years ago, enhances periodontal regeneration by mimicking the development of periodontal attachment tissues. Long-term follow-up clinical studies show that treatment of deep periodontal intrabony defects with enamel matrix derivative (EMD) significantly improved clinical attachment gain and intrabony fill compared to open flap debridement [12–14]. Iorio Siciliano et al. demonstrated in a seminal long-term reentry study that treatment of periodontal intrabony defects with enamel matrix derivative (EMD) leads to sustained improvements in clinical attachment and probing depths, with complete defect resolution in most sites [15]. Strong evidence supports the use of EMD both alone and in combination with bone grafts, as demonstrated by Iorio Siciliano et al., who reported comparable outcomes between EMD and deproteinized bovine bone mineral (DBBM) and grafting with a collagen membrane [16], while Matarasso et al. reported that adjunctive bone grafting may provide modest additional benefit [17]. Furthermore, Windisch et al. demonstrated that EMD-treated defects heal effectively regardless of flap design, with minimally invasive and extended papilla-preserving flaps producing similar improvements in clinical parameters [18]. Recent studies have expanded regenerative strategies for the management of intrabony periodontal defects beyond enamel matrix derivative. In a 6-month randomized controlled trial, Vela et al. demonstrated that cross-linked hyaluronic acid (xHyA) yielded clinical and radiographic outcomes comparable to EMD, indicating its potential as an alternative regenerative approach [19]. These studies have highlighted the consistent effectiveness and versatility of EMD as a regenerative therapy for intrabony defects.

Findings of a randomized, controlled clinical trial comparing treatment of deep intrabony defects with a new generation of PRF or EMD have shown that after 6 months both treatments resulted in significant clinical improvements evidenced by probing depth (PD) reduction and gain in clinical attachment level (CAL) without, however, any significant differences between the two groups [20]. Currently, long-term evaluations of regenerative periodontal surgery using PRF are limited, and to the best of our knowledge, no long-term outcome data exist comparing A-PRF+ with EMD.

The aim of the study was to investigate the long-term effects of human autologous platelet concentrates on periodontal healing and regeneration.

## Materials and methods

The null hypothesis of the study was whether an autologous material (A-PRF+) can be a reliable long-term alternative in healing of intrabony defects.

### Study design

The study was conducted at the Department of Periodontology, Semmelweis University, Budapest, Hungary. It was initiated in June 2018 and completed in November 2019 by the same experienced periodontist (BKCsN) [20]. The long-term evaluation (five years) took place in autumn 2024. This study was planned as a randomized, controlled, clinical trial, performed in accordance with the Helsinki Declaration of 1975, as updated in 2013 [21], and the protocol was approved by the Ethics Committee of the Semmelweis University Budapest (SE TUKEB: 254/2017) [20]. The study protocol was retrospectively registered at ClinicalTrials.gov with ID number NCT04404374. All participants received explanations of risks, benefits, and procedures in their native language and provided written informed consent.

### Study population

Study population and intrabony defect distribution are presented in Table 1 and 2. All patients were classified into stage III. periodontitis [22]. At baseline, patients received cause-related periodontal therapy (oral hygiene instruction, motivation, subgingival scaling/root planing under local anesthesia).

**Table 1:** Distribution of the study population

	Baseline	6 months postop.	5 years postop.
Male	9	9	6
Female	9	9	8
Total	18	18	14
Intrabony defects	30	30	26

Patients were consecutively enrolled upon meeting the following inclusion criteria [20]: (1) no systemic diseases; (2) good level of oral hygiene with Full-Mouth Bleeding Score < 20% [1] and Full- Mouth Plaque Index Score < 20% [23]; (3) the presence of one intrabony defect with a PD of ≥ 6 mm and an intrabony component of ≥ 4 mm as detected on radiographs, with a defect angle of 20–40 (+/- 5) degrees [24]; (4) no smoking [25, 26]. Exclusion criteria included systemic conditions potentially affecting healing (e.g. diabetes mellitus, osteoporosis, immunosuppression), poor oral hygiene, smoking, furcation involvement, and one-wall intrabony defects.

### Clinical parameters

The following clinical parameters were evaluated at baseline (1 week preoperatively), as well as at 6 months [20] and at 1 and 5 years postoperatively: using the same type of periodontal probe (UNC-15, Hu-Friedy, Chicago, IL, USA): Full-Mouth Plaque Score (FMPS) [23] and Full-Mouth Bleeding Score (FMBS) [1], pocket depth (PD), gingival recession (GR), clinical attachment level (CAL) and transgingival bone sounding (BS). The primary outcome was CAL. Probing depths were measured at six sites per tooth (mesio-/mid-/disto-buccal and lingual), and the highest PD value was taken into consideration [20]. Radiographs (long-cone technique) were taken preoperatively and 6 months, 1 and 5 years postoperatively. As no separate radiographic assessment was conducted, radiographic image standardization was not performed.

### Blinding and calibration

The examiner (ES, then during the 5-year control BCZ) was not aware, in any of the cases, of the type of treatment rendered. The examiners were blinded to the treatment type in all cases.

Examiners calibration involved five patients who were not enrolled in the study, each with 10 teeth (single- and multirooted) exhibiting probing depth > 6 mm on at least one site per tooth. The examiner assessed these patients twice, 48 h apart. Calibration was achieved if > 90% of recordings were reproducible within a 1.0 mm difference.

### Randomization

Prior to surgery, defects were randomly assigned using a computer-generated randomization sequence (https://www.sealedenvelope.com) to either the A-PRF+ (test group) or EMD (control group) [20] in a 1:1 allocation ratio. Simple randomization was applied without blocking or stratification. The allocation sequence was generated and stored electronically within the secure web-based platform and was not accessible to the operator prior to assignment. Defects were enrolled consecutively, and treatment allocation was disclosed only after enrollment and immediately before the surgical intervention, thereby ensuring allocation concealment and minimizing the risk of selection bias. The defects were distributed in a homogeneous configuration between the two groups (Table 2).

**Table 2:** Distribution and configuration of treated defects after 5 years

	A-PRF+	EMD
Tooth Location		
Maxilla	6	7
Mandible	7	6
Anterior teeth	3	2
Premolars	6	5
Molars	4	6
Defect Configuration		
2-wall	3	2
3-wall	4	4
2-3-wall combined	6	7

### Preparation of A-PRF+

Immediately before surgery, A-PRF + was prepared for the test group using a commercially available PRF Kit [Process for PRF^®^ (A-PRF), J. Choukroun, Nice, France] and a ‘Process for PRF Duo’ centrifuge (Choukroun). Cubital venous blood was drawn without anticoagulant into two 10 ml A-PRF+ tubes and immediately centrifuged at 1300 rpm for 8 min, followed by a 5-minute rest [6]. The PRF clot, while still in gel form, was removed from the tube, cleaned of red blood cells, and applied as a gel [20].

### Surgical procedure

**Fig. 1:**
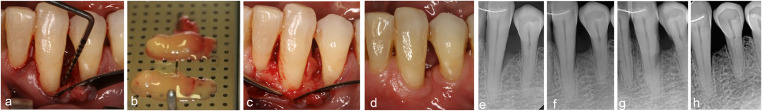
Treatment of an intrabony defect at a lower jaw incisor with A-PRF+. **a** defect after debridement - intraoperative measurements. **b** product after centrifugation in the PRF Box (Process for PRF®). **c** intrabony defect filled with A-PRF+. **d** 5 years after surgery. **e** - **h** radiographic evaluation before, 6 months, 1 and 5 years after surgery

**Fig. 2:**
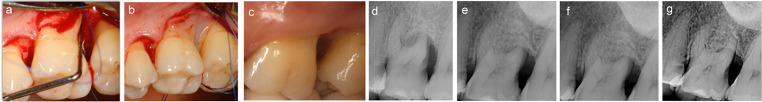
Treatment of an intrabony defect at an upper jaw molar with EMD. **a** defect after debridement – intraoperative measurements. **b** EMD applied in the intrabony defect. **c** 5 years post-operatively. **d** - **g** radiographic evaluation before, 6 months, 1 and 5 years after surgery

Surgery was performed by a single experienced periodontist (BKCSN). Following local anesthesia, intracrevicular incisions were performed, extending to adjacent teeth, with careful preservation of interdental gingival tissue. Full-thickness buccal and oral extended flaps were elevated, and all granulation tissue was removed from the defect without bone recontouring [20]. After defect debridement, A-PRF + was applied in the test group (Fig. 1). In the control group, following debridement, the root surface adjacent to the defect was conditioned for 2 min with 24% EDTA gel (pH 6.7) (PrefGel, Straumann^®^, Basel, Switzerland) [27]. The defect and adjacent mucoperiosteal flap were then thoroughly rinsed with sterile saline to remove all EDTA residue, followed by the application of Straumann^®^ Emdogain^®^ (Fig. 2). Finally the flaps were repositioned coronally and securely closed using 5.0 nonabsorbable modified vertical or horizontal mattress sutures [20].

### Postoperative care

All patients received antibiotics for one week, twice daily (Augmentin Duo, 875 mg amoxicillin/125 mg clavulanic acid, GlaxoSmithKline, Brentford, United Kingdom) [28]. Postoperative care included 0.2% chlorhexidine rinses (Curasept ADS 220, Curaden AG, Kriens, Switzerland) twice daily for three weeks. Patients were instructed to avoid mechanical plaque control in the surgical area for three weeks. Sutures were removed 14 days after surgery, and proper oral hygiene maintenance was reinforced [20]. Participants were scheduled for follow-up visits weekly for one month after surgery, followed by visits at three- and six-month intervals. Over the subsequent four years, patients underwent supportive periodontal therapy (SPT) with recall visits scheduled every three to six months based on individual needs. During each SPT visit oral hygiene practices were reinforced, supragingival and subgingival scaling, and polishing was performed.

### Statistical analyses

Clinical parameters were analyzed using descriptive statistics, with results reported as mean ± standard deviation (SD) and range at baseline, 6-months [20], 1 year and 5 years intervals. Data handling and analysis were performed using Stata (StataCorp. 2017. Stata Statistical Software: Release 15. College Station, TX: StataCorp LLC). Defects, rather than patients, were used as the unit of observation. 5 years postoperatively, two samples of 13 observations were adequately powered (80%) to detect a between-groups difference of 1.15 standard deviations (SD) in a continuous variable, assuming equal SDs across groups.

Post-hoc power calculations were conducted for between-group comparisons at 5 years and within-group comparisons (5 years vs. baseline) for each outcome. Within-group changes were assessed using paired t-tests or Wilcoxon’s matched-pairs signed-ranks test if parametric assumptions were not met. Between-group comparisons were performed using two-sample t-tests or Wilcoxon’s rank-sum test if parametric assumptions were not satisfied. P-values < 0.05 were considered statistically significant. Changes in frequency distribution from baseline to 5 years were categorized as decrease, no change, or increase for each outcome, and between-group differences were evaluated using Fisher’s exact test (Table 3).

**Table 3:** Frequency distribution of the results 6 months and 5 years postsurgically (intergroup comparison)

	A-PRF+		EMD		Total	
PD	6 months	5 years	6 months	5 years	6 months	5 years
decrease	15	13	15	13	30	26
total	15	13	15	13	30	26
GR						
decrease	0	0	1	2	1	2
no change	5	4	4	4	9	8
increase	10	9	10	7	20	16
total	15	13	15	13	30	26
Fisher’s exact					1.000	0.580
CAL						
decrease	13	12	15	13	28	25
no change	2	1	0	0	2	1
total	15	13	15	13	30	26
Fisher’s exact					0.483	1.000
BS						
decrease	14	13	14	13	28	26
no change	1	0	1	0	2	0
total	15	13	15	13	30	26
Fisher’s exact					1.000	1.000

*PD* probing depth, *GR* gingival recession, *CAL* clinical attachment level, *BS* bone sounding

## Results

**Fig. 3:**
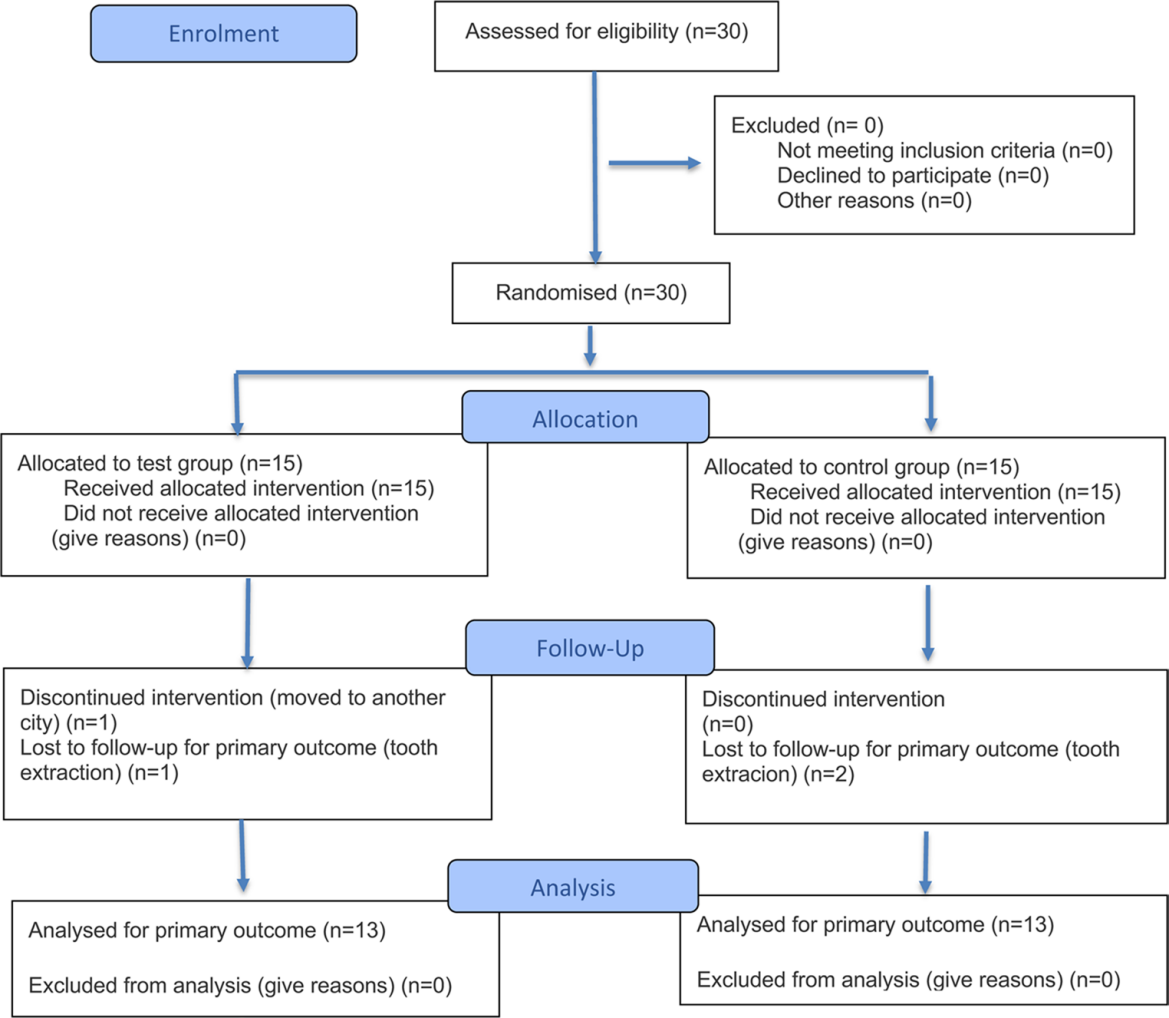
Flow diagram of patient enrollment and study process

The study population, the surgical and postoperative protocol, and the 6 months results have been described previously [20]. Twenty-six (26) intrabony defects (6 males and 8 females, aged 55.5 +/- 14.5 years at baseline, 13 intrabony defects in each group) completed the 5-year evaluation (Table 1 and 2). The study flowchart is presented in Fig. 3. Baseline and postoperative (6 months and respectively 5 years) full-mouth bleeding scores (FMBS) and full-mout h plaque scores (FMPS) were comparable, with FMBS values decreasing after surgery [20]. The 5-year values were similar to the 6 months results. The findings on early postoperative healing have been reported previously [20].

No statistically significant differences were found between the two groups regarding the mean values of the baseline clinical parameters. After 5 years, the mean PD has decreased significantly in both groups compared to baseline data (*p* < 0.001). The mean PD reduction was 3.92 ± 0.75 mm in the A-PRF+ group and similarly 3.92 ± 1.03 mm in the EMD group. Upon intergroup comparison no statistically significant difference was found (Table 4).

**Table 4:** Within group comparisons / intergroup changes after 5 years (significance: *p* < 0.05)

	Baseline	5 years postop.	*p*	Diff.	Cohen’s d
PD					
A-PRF+	8.23 ± 1.58	3.92 ± 0.75	*p* < 0.0001 (s.)	-4.3 ± 1.79	-2.40
EMD	8.38 ± 1.55	3.92 ± 1.03	*p* < 0.0001 (s.)	-4.46 ± 1.39	-3.21
		*p* > 0.999 (n.s.)			
GR					
A-PRF+	2.53 ± 1.89	3.92 ± 2.81	*p* = 0.0048 (s.)	1.38 ± 1.44	0.96
EMD	2.07 ± 1.11	2.76 ± 0.72	*p* = 0.056 (n.s.)	0.69 ± 1.18	0.59
		*p* = 0.34 (n.s.)			
CAL					
A-PRF+	10.76 ± 2.97	7.84 ± 2.76	*p* < 0.0001 (s.)	-2.92 ± 1.65	-1.76
EMD	10.46 ± 1.85	6.61 ± 1.44	*p* < 0.0001 (s.)	-3.84 ± 1.81	-2.11
		*p* = 0.171 (n.s.)			
BS					
A-PRF+	9.46 ± 1.66	4.92 ± 0.64	*p* = 0.0002 (s.)	-4.53 ± 1.66	-2.73
EMD	9.76 ± 1.58	4.76 ± 1.09	*p* < 0.0001 (s.)	-5 ± 1.58	-3.16
		*p* = 0.665 (n.s.)			

*PD* probing depth, *GR* gingival recession, *CAL* clinical attachment level, *BS* bone sounding

5 years postoperatively, the GR increased with 1.38 ± 1.44 mm in the test group and with 0.69 ± 1.18 mm in the control group. The increase in GR was statistically significant for the test group (*p* = 0.0048), but no difference between the groups was observed (Table 4).

The mean CAL gain was 2.92 ± 1.65 mm in the A-PRF+ group and 3.84 ± 1.81 mm in the EMD group (*p* < 0.001). In both groups, the CAL has improved significantly compared to baseline, but no statistically significant difference was found upon intergroup comparison (Table 4).

After 5 years, the mean BS has decreased to 4.92 ± 0.64 mm in the test group and to 4.76 ± 1.09 mm in the control group. Compared to baseline data 9.46 ± 1.66 mm in the test group, and 9.76 ± 1.58 mm in the control group, the mean BS reduction was significant (*p* < 0.001), but no difference between the groups was observed, the results obtained with both materials were similar (Table 4).

Following surgical intervention, similar values were observed in both groups after 1and 5 years in terms of the investigated parameters. A further, but not significant, decrease in probing depth (PD), along with a further reduction in bone sounding (BS) and improvement in clinical attachment level (CAL) values, can be observed between first and fifth year (Table 5). Although both A-PRF + and EMD continued to show similar effects more years after surgery, the degree of improvement in the measured parameters was lower than that observed during the initial six months.

**Table 5:** Within group changes between 1 and 5 years (significance: *p* < 0.05)

	1 year	5 years	Diff.	*p*	Cohen’s d
PD					
A-PRF+	4.61 ± 1.26	3.92 ± 0.75	-0.69 ± 1.60	*p* = 0.171 (n.s.)	-0.43
EMD	4.23 ± 0.59	3.92 ± 1.03	-0.30 ± 0.94	*p* = 0.264 (n.s.)	-0.32
GR					
A-PRF+	4.07 ± 2.66	3.92 ± 2.81	-0.15 ± 1.14	*p* = 0.636 (n.s.)	-0.13
EMD	2.61 ± 1.66	2.76 ± 0.72	0.15 ± 1.51	*p* = 0.721 (n.s.)	0.10
CAL					
A-PRF+	8.69 ± 2.89	7.84 ± 2.76	-0.84 ± 2.19	*p* = 0.304 (n.s.)	-0.39
EMD	6.84 ± 2.07	6.61 ± 1.44	-0.23 ± 1.53	*p* = 0.597 (n.s.)	-0.15
BS					
A-PRF+	5.30 ± 1.43	4.92 ± 0.64	-0.38 ± 1.70	*p* = 0.683 (n.s.)	-0.22
EMD	4.92 ± 0.64	4.76 ± 1.09	-0.15 ± 0.80	*p* = 0.501 (n.s.)	-0.19

*PD* probing depth, *GR* gingival recession, *CAL* clinical attachment level, *BS* bone sounding

Post-hoc power calculations for between-group comparisons of probing depth (PD) could not be estimated due to identical group means at 5 years. For gingival recession (GR), clinical attachment level (CAL) and bone sounding (BS) values, the estimated power was 26.6%, 27.1%, and 7%, respectively. For within-group comparisons, the estimated power for gingival recession (GR) was 96.7%, and power estimates of 99.9% were achieved for all other outcomes.

## Discussion

The present study demonstrated that both autologous A-PRF + and xenogeneic EMD resulted in significant clinical improvements in the treatment of periodontal intrabony defects. At 6 months a mean probing depth (PD) reduction of 4.67 ± 0.62 mm and a clinical attachment level (CAL) gain of 2.33 ± 1.58 mm was achieved with A-PRF+ [20]. At the 5-year follow-up, further improvement was observed, with PD reduction reaching 3.92 ± 0.75 mm and CAL gain increasing to 2.92 ± 1.65 mm. Compared with baseline values, PD and BS decreased to a similar extent in both groups 5 years postoperatively. The rate of reduction was more pronounced during the first six months than in the second half of the healing period. However, the continued improvement observed even 5 years postsurgically suggests that the filling of intraosseous defects progresses beyond the initial six months, as the maturation and mineralization of periodontal hard tissues require a longer period.

**Fig. 4:**
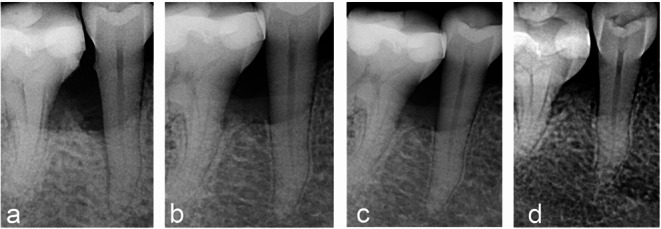
Treatment of an intrabony defect at a lower jaw premolar with A-PRF+. **a** preoperative radiograph. **b** 6 months after surgery partial fill of the intrabony component. **c** at 1 year defect fill is clearly visible. **d** at 5 years complete defect fill and stability

**Fig. 5:**
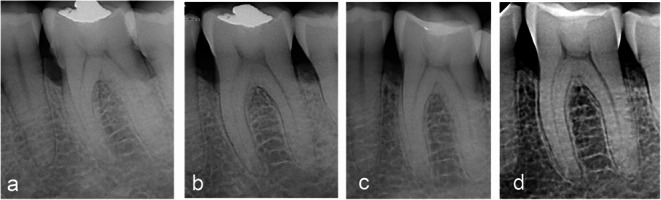
Treatment of an intrabony defect at a lower jaw molar with EMD. **a **preoperative radiograph. **b** 6 months post-surgically partial fill of the intrabony component. **c** at 1 year defect fill is clearly visible. **d** at 5 years complete defect fill and stability

In addition to the substantial improvement in transgingival probing values (BS), intraoral radiographs taken using the long-cone technique demonstrated radiographic evidence of bone fill. When evaluating healing, it is important to consider that hard-tissue formation and maturation require more time than soft-tissue healing and may vary considerably between individuals. Based on our experience, radiographic detection of newly formed, mature, mineralizing bone tissue often requires at least one year (Figs. 4 and 5), [29].

Following surgical therapy and resolution of inflammation, gingival recession increased significantly in both groups compared with baseline. However, the pronounced reduction in probing depth resulted in a significant gain in clinical attachment level.

Beyond the resolution of intraosseous defects, both treatment modalities yielded additional clinical benefits, most notably significant pocket depth reduction, thereby facilitating long-term mechanical plaque control. Upon healing, re-establishment of periodontal homeostasis may provide stable, inflammation-free conditions, provided that adequate plaque control and regular supportive periodontal care are maintained [30, 31].

Advances in cell and molecular biology have significantly enhanced our understanding of wound-healing mechanisms. It has been demonstrated that polypeptide-derived growth factors can support wound healing and tissue regeneration by promoting chemotaxis, cellular differentiation and proliferation, as well as regulating matrix synthesis [32].

As a second-generation platelet concentrate, PRF has become widely accepted across numerous fields of regenerative medicine [33–35], including periodontal surgery, where its regenerative potential has been evaluated in several randomized clinical trials and summarized in recent meta-analyses [36, 37]. Following centrifugation, the dense, leukocyte-rich preparation supports the repair of damaged tissues through its bio-organic fibrin matrix. An increasing number of studies highlight the beneficial effects of leukocytes on healing and tissue regeneration, as well as the importance of fibrin network quality. It has been established that PRF possesses the antimicrobial and immunoregulatory functions of leukocytes [38] and is characterized by substantial VEGF release [39].

In recent years, the outcomes of studies evaluating the effects of PRF in the treatment of periodontal intrabony defects have been summarized in a meta-analysis [36]. Compared with open flap debridement (OFD), the potentiating effect of PRF was observed across 14 randomized, controlled clinical trials. On average, after approximately 8 months of application, probing depth (PD) was reduced by 1.3 mm, while clinical attachment level (CAL) increased by an average of 1.5 mm [40–42]. In our clinical study, following the application of A-PRF+, a mean PD reduction of 4.67 ± 0.62 mm and a CAL gain of 2.33 ± 1.58 mm were already observed at 6 months [20]. Compared with the values reported in the meta-analysis, A-PRF+ yielded more favorable clinical outcomes. At the 5-year follow-up, these values demonstrated additional improvement.

It has been demonstrated that the biological benefits of PRF are mediated through its effects on the proliferation and differentiation of various cell types [43]. Based on the available literature, PRF appears to influence soft-tissue regeneration more effectively than hard-tissue regeneration [43]. According to the systematic literature review, in vitro studies highlight the positive role of PRF in promoting endothelial cell proliferation and angiogenesis.

Modification of the preparation protocol for platelet-rich fibrin (PRF), specifically by reducing the applied relative centrifugal force (RCF) to 208 g, has resulted in a seemingly more favorable technique. Compared with conventional PRF, the A-PRF clot exhibits a less dense structure with larger interfibrillar spaces, within cells - particularly platelets - are more evenly distributed throughout the entire fibrin matrix. Furthermore, histological analysis of A-PRF has revealed a significantly higher number of neutrophil granulocytes [8]. Further refinement of the centrifugation protocol led to the development of an advanced platelet concentrate with even more advantageous characteristics, referred to as Advanced PRF Plus (A-PRF+) [6].

Recent findings are supported by a comparative study that evaluated the effect of A-PRF + on the healing of intraosseous defects in comparison with open flap debridement [44]. In this clinical trial involving 22 patients, the test group received open flap debridement combined with A-PRF+, whereas the control group was treated with open flap debridement alone. Clinical outcomes were recorded at 3, 6, and 9 months postoperatively. Significant reductions in probing depth (PD) and significant gains in clinical attachment level (CAL) were observed in the test group compared with the control group. The amount of PD reduction was comparable to the values recorded in our clinical study as well as to those reported in our published case series [29] (6 months: 3.64 ± 1.12 mm; 9 months: 3.73 ± 1.19 mm). However, the extent of CAL gain (6 months: 3.36 ± 1.12 mm in the test group vs. 2.36 ± 0.81 mm in the control group) differed from the attachment improvements observed in our own clinical investigation. In our control group (treated with EMD), we recorded a greater increase in CAL at 1-year post-surgery (3.46 ± 1.59 mm), whereas the test group (A-PRF+) demonstrated a lower gain (2.6 ± 2.84 mm). The observed discrepancy may be attributable to differences in baseline GR values.

Stability of the coagulum is crucial during periodontal wound healing. Owing to its favorable biological properties, PRF serves as a biocompatible matrix that supports postoperative healing, which has contributed to its growing popularity in periodontal surgical procedures. Since both the surgical technique and the method of flap design can significantly influence treatment outcomes, further studies are warranted to determine the optimal therapeutic approach [36]. According to the most recent therapeutic guidelines endorsed by the European Federation of Periodontology (EFP), the recommended treatment for periodontal intraosseous defects includes the use of EMD or a resorbable membrane in combination with a papilla preservation flap design [45].

Enamel matrix derivatives (EMD) remain one of the most well-documented adjuncts for periodontal regenerative surgery, with evidence showing their effectiveness in reducing PD and improving CAL over extended follow-up periods, including up to 10 years when combined with bone grafting [46–48]. Our control group outcomes were consistent with these reports, confirming the long-term stability and clinical relevance of EMD-based therapy. Nonetheless, anatomical factors such as defect configuration can influence regenerative outcomes [49]. Complete and predictable periodontal regeneration remains challenging, particularly in advanced intrabony defects. Both surgical technique and flap design significantly influence treatment outcomes. In the present study, two biologically active materials with comparable physical handling characteristics were evaluated using open flap debridement, allowing assessment of their relative clinical performance.

Despite the favorable outcomes observed, several limitations must be acknowledged. The study was designed as a two-arm trial without a separate untreated control group, and direct comparisons between A-PRF + and EMD remain limited in the literature. The biological variability of intrabony defect healing may influence outcomes [50]. Although the long-term follow-up supports the clinical relevance, the relatively small sample size limits the strength of the conclusions. The absence of differences between groups may reflect the limited number of treated defects, with an estimated 30 patients per group required for adequately powered superiority trials in the management of periodontal intrabony defects [51]. Furthermore, while short-term data on A-PRF and A-PRF + are increasing, long-term clinical evidence beyond 12 months remains scarce. Prospective, well-designed trials with larger sample sizes are necessary to determine the sustained efficacy of A-PRF + and to clarify its relative benefits compared with conventional PRF and enamel matrix derivatives. Nevertheless, the present long-term findings suggest that an autologous material (A-PRF+) may represent a promising adjunct in periodontal regenerative surgery and open new opportunities for optimizing regenerative protocols.

## Conclusion

New-generation platelet-rich fibrin (A-PRF+) seems to be as effective as enamel matrix derivative (EMD) in the surgical treatment of intrabony periodontal defects. The clinical outcomes achieved with both treatments were maintained for up to 5 years. The autologous A-PRF+ appears to be a suitable treatment option for intrabony periodontal defects.

## Data Availability

Authors can confirm that all relevant data are included in the article and/or its supplementary information files.

## Abbreviations

A-PRF: advanced platelet-rich fibrin
EMD: enamel matrix derivative
PRP: platelet-rich plasma
PRG: platelet-rich gel
PRF: platelet-rich fibrin
PRGF: plasma rich in growth factors
L-PRF: leukocyte- and platelet-rich fibrin
VEGF: vascular endothelial growth factor
NBM: natural bone mineral
DBBM: deproteinized bovine bone mineral
xHyA: cross-linked hyaluronic acid
PD: probing depth
GR: gingival recession
CAL: clinical attachment level
BS: bone sounding
FMBS: full-mouth bleeding score
FMPS: full-mouth plaque score
EDTA: ethylenediamine tetraacetic acid
SPT: supportive periodontal therapy

## Declarations

All the included patients signed an informed consent which was previously approved by an institutional ethics committee.
